# Primary pleomorphic adenoma of minor salivary gland in the parapharyngeal space

**DOI:** 10.1186/1477-7819-7-85

**Published:** 2009-11-12

**Authors:** Arsheed H Hakeem, Biswajyoti Hazarika, Sultan A Pradhan, Rajan Kannan

**Affiliations:** 1 Department of Surgical Oncology, Prince Aly Khan Hospital, Aga Hall, Nsbit Road, Mazagaon, Mumbai, India

## Abstract

**Background:**

World literature suggests parapharyngeal space lesions account for only 0.5% head and neck tumours and the majority of the minor salivary gland tumours are malignant. The parapharyngeal space is very rare site for this tumour.

**Case presentation:**

Two cases of primary pleomorphic adenomas arising *de novo *from minor salivary glands in the para pharyngeal space are reported. Review of literature, clinical features, pathology, radiological findings and treatment of these tumours are discussed.

**Conclusion:**

Pleomorphic adenoma arising de novo in the parapharyngeal space is of rare occurrence. High index of suspicion and an adequate clearance of the tumour with a cuff of surrounding dispensable normal tissues is the key to successful treatment of such tumours.

## Background

Parapharyngeal space tumours are not very frequent, accounting for some 0.5% of neoplasms of head and neck. Most of these tumours (70%-80%) are benign and 40-50% of these originate in the salivary glands, particularly the pleomorphic adenoma [1]. Pleomorphic adenoma in the parapharyngeal space (PPS) can develop *de novo *or may arise from deep lobe of the parotid and extend through the stylomandibular tunnel into the PPS [2]. The origin of *de novo *pleomorphic adenoma is probably from displaced or aberrant salivary gland tissue within a lymph node [3]. However, pleomorphic adenoma arising *de novo *in the parapharyngeal space is extremely rare which made us to report these cases.

## Case presentation

### Case 1

**Figure 1:**
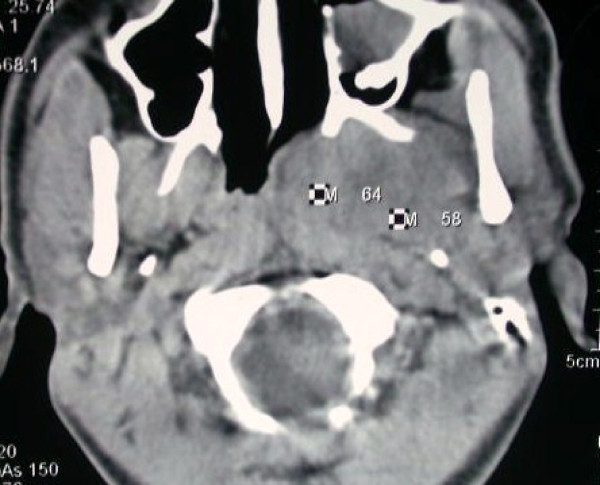
**Axial CT scan showing homogenously enhancing lesion**.

**Figure 2:**
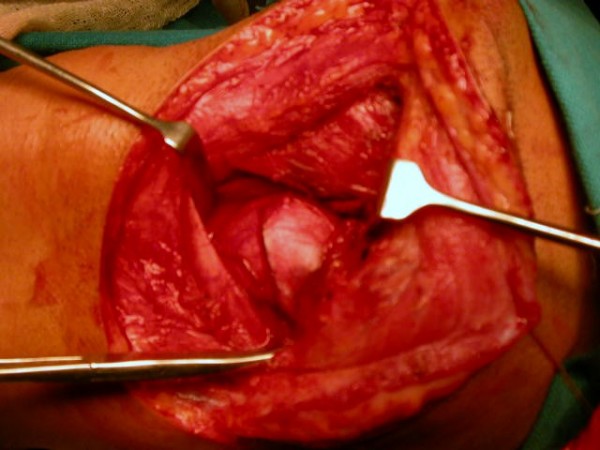
**Access gained to parapharngeal space through neck**.

A 20 -year- old male presented with gradually progressive painless swelling of the left upper neck and change in the quality of voice of 1 year duration. On intraoral examination there was a smooth firm bulge of the soft palate and left lateral pharyngeal wall (Figure 1). Neck examination revealed a firm swelling in the upper neck involving retromandibular region on the left side. There was no history of difficulty in swallowing. The swelling was bimanually palpable and ballotable. Posterior nasal examination showed the extension of the swelling into the nasopharynx. There was no significant lymph node enlargement in the neck. Clinical examination did not reveal involvement of any of the cranial nerves. With a clinical diagnosis of parapharyngeal space tumour a CT scan was taken which showed homogenously enhancing tumour measuring 7 × 6 cm in the left parapharyngeal space, extending from skull base to the hyoid bone (Figure 2). Fine needle aspiration cytology was consistent with benign mixed tumour of salivary gland origin.

**Figure 3:**
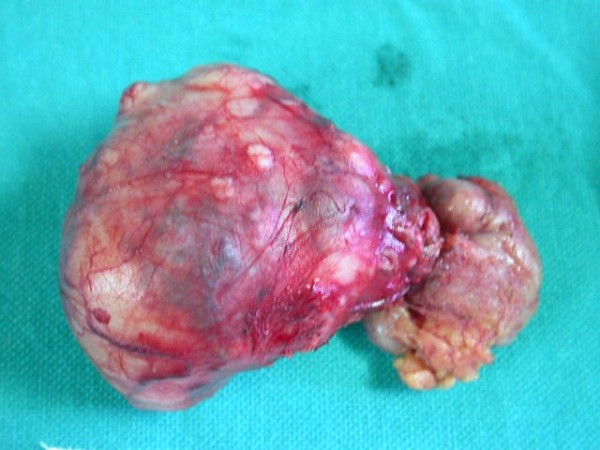
**Surgical specimen**.

**Figure 4:**
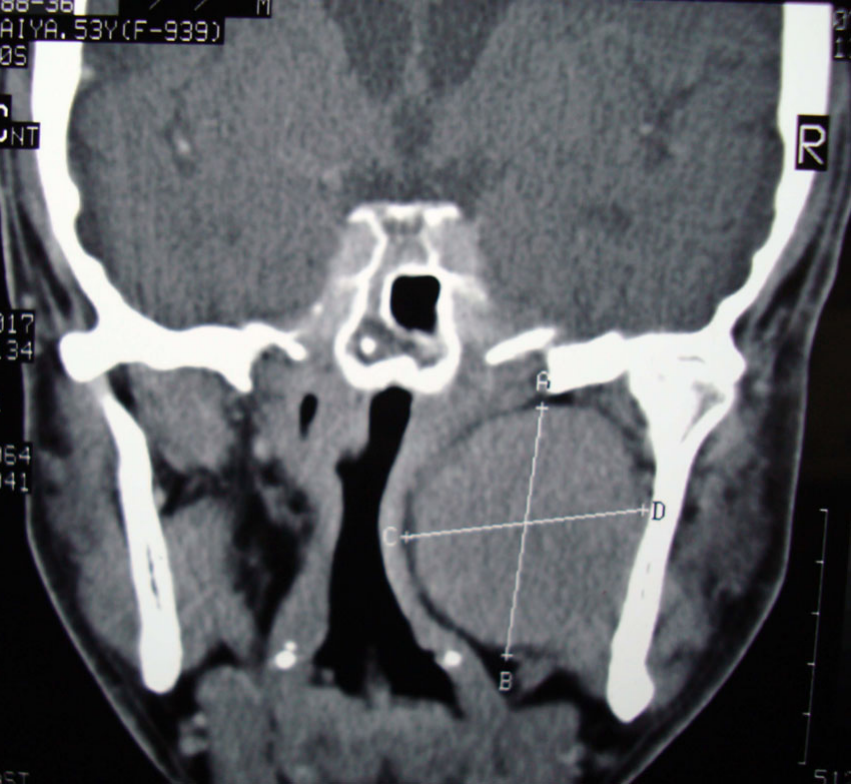
**Post contrast coronal CT scan showing parapharngeal lesion**.

Trans- cervical approach was used to gain access to the left parapharyngeal space (Figure 3), the tumour was completely excised. On gross examination the lesion was 8 × 6 cm with a whitish, lobulated and glistening surface (Figure 4). Histopathological examination showed a neoplasm having an admixture of epithelial and stromal components. Ducts lined by inner epithelial and outer myoepithelial cells were seen surrounded by a chondromyxoid stroma consistent with pleomorphic adenoma. Postoperative period was uneventful. Patient is free of disease after a period of 2 years.

### Case 2

**Figure 5:**
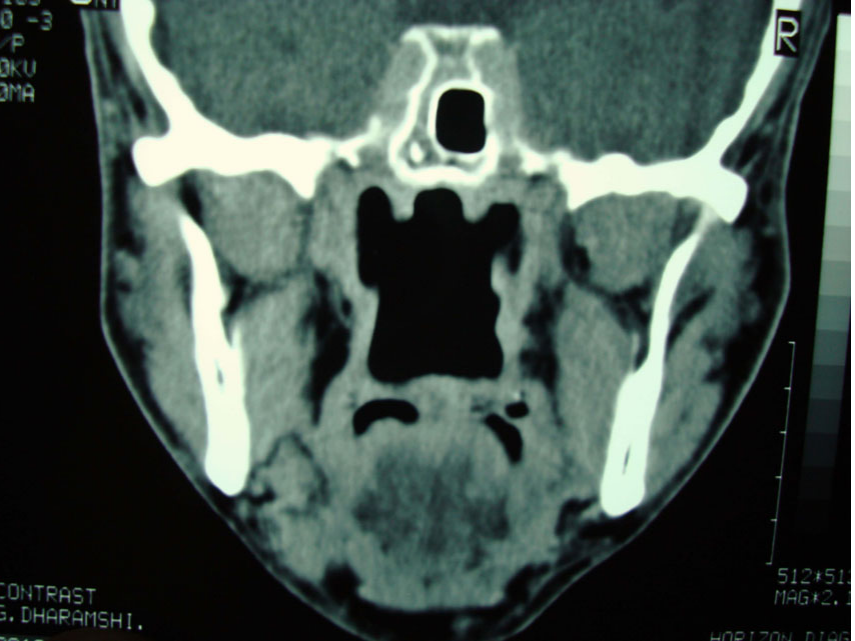
**Post contrast CT of the same patient**.

A 53-year- old male presented with history of change in voice with foreign body sensation in the throat. A physical examination showed right intraoral mass displacing the soft palate medially. On careful neck palpation a firm swelling was palpable in the right upper neck. The swelling was bimanually palpable and ballot able. Posterior nasal examination showed extension of the swelling into the nasopharynx and indirect laryngoscopy revealed the lower limit of swelling at the level of valeculla. There was no significant lymph node enlargement in the neck. With the clinical diagnosis of parapharyngeal tumour a CT scan of the head and neck was taken which showed a well defined 6 × 5 cm mass occupying the right parapharyngeal space with homogenous contrast enhancement (Figure 5). After fine needle aspiration cytology it was diagnosed as pleomorphic adenoma.

Through a right transverse neck incision, entry was gained to para pharyngeal space, the tumour was excised completely. On gross examination the lesion was 6 × 5 cm in size with a whitish lobulated and focally glistening cut surface. Histological examination showed a neoplasm having an admixture of epithelial and stromal components. Ducts lined by inner epithelium and outer myoepithelial cells were seen surrounded by a chrondromyxoid stroma consistent with pleomorphic adenoma. Post operative period was uneventful. Repeat CT scan done after 3 years of follow up does not show any evidence of residual or recurrent disease.

## Discussion

Tumours arising in the minor salivary glands account for 22% of all salivary gland neoplasms [4]. Majority of them are malignant with only 18% being benign. Of all the benign tumours pleomorphic adenoma is the commonest [4]. The most common site of pleomorphic adenoma of the minor salivary glands is the palate followed by lip, buccal mucosa, floor of mouth, tongue, tonsil, pharynx, retro molar area and nasal cavity [4-7]. Pleomorphic adenoma of the parapharyngeal space is rare. *De novo *occurrence of the pleomorphic adenoma in our patients can arise from displaced or aberrant salivary gland tissue within a lymph node in the parapharyngeal space as suggested by Varghese et al [3].

Another source of such tumour is deep lobe of parotid gland, in which case the tumour may present as a dumb bell tumour abutting the stylohoid ligament [8]. A comprehensive review of literature showed very few case reports of pleomorphic adenoma arising *de novo *in the parapharyngeal space [3].

Though most of the benign tumours of the minor salivary gland in the oral cavity present as a painless submucosal swelling [4], those from the parapharyngeal space may show additional symptoms, like otalgia, neuralgia, palsies of 9^th^, 10^th^, or 11^th ^cranial nerves or trismus. Classical findings of benign parapharyngeal swelling are a submucosal swelling in the lateral pharyngeal wall with or without extension to retromandibular fossa or the submandibular trigone and bimanual ballot ability [8-10].

CT scan and MRI are important diagnostic tools in tumours of parapharyngeal space. These help in determining the extent of disease, local spread and also help to some extent in determining the type of tumour. Contrast enhancement is seen in vascular and neurogenic tumours. Presence of intact fat plane helps in distinguishing benign tumours from malignant ones. Extension of tumours from the deep lobe of a parotid gland is distinguishable from tumour arising de novo in parapharyngeal space by a fine translucent line representing the compressed layer of fibroadipose tissue between the tumour and deep lobe of parotid [11]. MRI has been shown to be superior to computed tomography in the investigation of parapharyngeal space tumours [12-14].

Fine needle aspiration cytology is the modality of choice for obtaining biopsy sample for diagnosis [2]. Incision biopsy is no more advocated for salivary gland tumour due to seeding of tumour and subsequent multinodular recurrence [2,15].

Histopathologically, pleomorphic adenoma is an epithelial tumour of complex morphology, possessing epithelial and myoepithelial elements arranged in a variety of patters and embedded in a mucopolysaccharide stroma. Formation of the capsule is a result of fibrosis of surrounding salivary parenchyma, which is compressed by the tumour and is referred to as "false capsule" [11].

The treatment of pleomorphic adenoma is essentially surgical [2,3,8,16]. Though these tumours are apparently well encapsulated, resection of the tumour with an adequate margin of grossly normal surrounding tissue is necessary to prevent local recurrence as these tumours are known to have microscopic pseudopod like extension into the surrounding tissue due to "dehiscences" in the false capsule [11]. The parapharyngeal space is however, a complex anatomic region located between the mandibular ramus and lateral pharynx and extending as an inverted pyramid from the skull base superiorly to hyoid bone inferiorly. Within this potential space are cranial nerves IX, X, XI, and XII, the sympathetic chain, carotid artery, the jugular vein and lymph nodes. Due to the PPS's anatomic complexity, location and surrounding vital structures, resection of tumours from this space can prove challenging to the head and neck surgeon. The approach of choice to the parapharyngeal space to allow adequate removal of the tumour should meet two criteria: wide intra-operative visibility for safe radical dissection and minimal functional and or cosmetic after-effects.

Traditionally, PPS surgery mainly uses the transcervical and parotid approaches. Malone *et al*. and Hamza *et al*. [17,18] describe the resection of PPS tumours using the transcervical approach alone in 90-100% cases. Hughes et al. [8] published a series of 172 cases using the transcervical and trans-parotid approaches in 94%, using mandibular osteotomy in only 2% of resections. The tran-soral approach described by Ehrlich [19] in 1950 is indicated for small, non vascular tumours, as it offers poor exposition and does not give adequate control in the event of haemorrhage. Works published by McElroth et al. [20] in 1963 describe the use of this approach along with ligature of external carotid artery to remove PPS tumours in a study on 112 patients. More recently, in 1989 Goodwin and Chandler [21] considered this approach to give adequate access to the PPS, as it gives direct access to the PPS. It is very useful combined with other techniques, as it allows the deepest part of the tumour to be exposed, allowing for the removal of larger tumours. The several kinds of mandibular osteotomies have been described in the literature to give excellent exposure. We prefer to use trans -oral approach in small tumours and a standard trans-cervical approach for large benign PPS tumours.

## Conclusion

Pleomorphic adenoma arising de novo in the parapharyngeal space is of rare occurrence. High index of suspicion and an adequate clearance of the tumour with a cuff of surrounding dispensable normal tissues is the key to successful treatment of such tumours.

## Consent

Written informed consent was obtained from both the patients for publication of this case report the copy of the consent is available with Editorial office.

## Competing interests

The authors declare that they have no competing interests.

## Authors' contributions

AH prepared the draft and literature search. BH helped in preperation of manuscript. SAP conceived the idea and edited the manuscript. RK was involved in preparation of manuscript.
